# Identification and verification of differentially expressed key genes in peripheral blood-derived T cells between chronic immune thrombocytopenia patients and healthy controls

**DOI:** 10.1080/21655979.2022.2080422

**Published:** 2022-06-06

**Authors:** Bingjie Ding, Liu Liu, Yingtong Dai, Mengjuan Li, Ao Xia, Xuewen Song, Jianping Liu, Xiaoran Wang, Yongping Song, Hu Zhou

**Affiliations:** a Department of Hematology, The Affiliated Cancer Hospital of Zhengzhou University & Henan Cancer Hospital, Hemostasis and Thrombosis Diagnostic Engineering Research Center of Henan Province, Zhengzhou, Henan, China; b Department of Hematology, The First Affiliated Hospital of Zhengzhou University, Zhengzhou, Henan, China; c Department of Blood Component Preparation, Henan Province Red Cross Blood Center, Zhengzhou, Henan, China

**Keywords:** Chronic immune thrombocytopenia, peripheral blood-derived T cells, bioinformatics analyses, clinical verification, key gene

## Abstract

Immune thrombocytopenia (ITP), characterized by decreased platelet counts, is a complex immune-mediated disorder with unelucidated pathogenesis. Accumulating evidence shows that T cell-mediated platelet destruction is one crucial process during the progression of ITP. Here, we attempted to identify core genes in peripheral blood-derived T-cells of chronic ITP through the analysis of microarray data (GSE43179) and clinical verification, with the aim to further understand the pathogenesis and progression of ITP. Compared with healthy controls, 97 differentially expressed genes (DEGs), including 63 up-regulated and 34 down-regulated were identified in ITP patients. Functional enrichment analysis showed that the DEGs were mainly enriched in innate immune response, inflammatory response, and IL-17 signaling pathway. Among the DEGs, top 15 hub genes ranked by degree score were identified via protein-protein interaction (PPI) network and were further confirmed by quantitative reverse transcription PCR (qRT-PCR). Among top 15 hub genes, the expression levels of 14 DEGs like *TLR4, S100A8, S100A9*, and *S100A12* were significantly up-regulated, while one DEG *IFNG* was down-regulated in ITP patients. Noticeably, *TLR4* exhibited the highest degree score, and *S100A8* had the largest fold change in qRT-PCR analysis. Altogether, our results suggested that the pathogenesis and progression of ITP are related with multiple immune-related pathways, and that *TLR4* and *S100A8* are likely to play crucial roles.

## Graphical abstract

**Figure:**
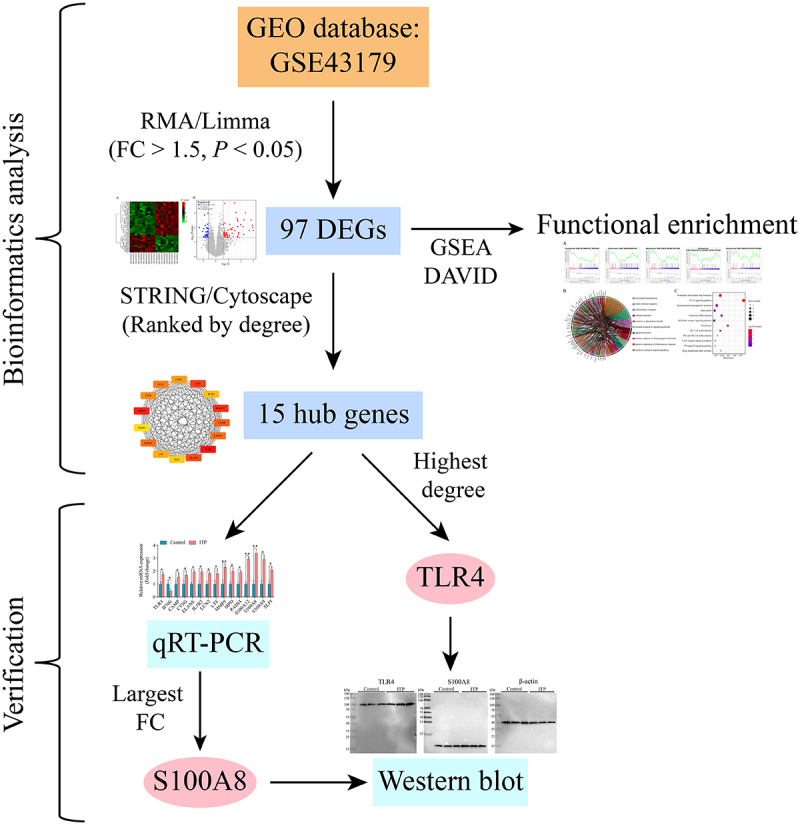

## Highlights

- A total of 97 DEGs were identified, including 63 up-regulated and 34 down-regulated
- The DEGs were related to innate immune response and IL-17 signaling pathway
- *TLR4* had the highest degree score and *S100A8* exhibited the highest fold change
- *TLR4* and *S100A8* play crucial roles in pathogenesis and progression of ITP.

## Introduction

Immune thrombocytopenia (ITP), an acquired autoimmune disorder can lead to transient or persistent decrease of the platelet counts (below 100 × 10^9^/L) and enhanced risk of bleeding [1,2]. The annual incidence of ITP is approximately 3.3 per 100,000 adults in Europe [3], and 6.1 per 100,000 persons in US [4], and this increases with age [5]. The disease causes the hemorrhage in skin and mucous membranes, internal organs, and even craniums, which seriously threatens human health [6]. At present, the pathogenesis of ITP remains unclear, but T cell-mediated platelet destruction is a key process during the progression of ITP [7]. Identification of differentially expressed key genes in peripheral blood-derived T cells between chronic ITP patients and healthy controls would be helpful for understanding the pathogenesis and progression of ITP.

Due to a simultaneous detection of multiple samples, extremely high sensitivity, and minimized systematic errors, bioinformatics analysis of microarray data has been widely used to identify key genes in various autoimmune diseases [8–10]. Through a comparative DNA microarray analysis of human joint fibroblast-like synoviocytes derived from rheumatoid arthritis and osteoarthritis, some key genes and pathways involved in the pathogenesis of rheumatoid arthritis, and osteoarthritis were identified [8]. With the aid of microarray datasets analysis, vitiligo-related biomarkers were identified [11]. Through bioinformatics analysis of microarray datasets and clinical verification, the hub gene *pro-ADM* was identified in male patients with gout [10]. However, the hub genes involved in the pathogenesis and progression of ITP were largely unexplored.

In the present study, to identify the differentially expressed key genes in peripheral blood-derived T cells between chronic ITP patients and healthy controls, an integration approach of bioinformatics analysis of microarray data downloaded from GEO dataset (GSE43179) and clinical verification was performed. The hub genes identified here would serve to better understanding for the pathogenesis and progression of ITP.

## Materials and methods

### Microarray data collection

The DNA microarray dataset (GSE43179) was collected from the Gene Expression Omnibus (GEO) database (http://www.ncbi.nlm.nih.gov/geo/) [12] based on a previous study [13]. The dataset comprised 19 samples, including 9 ITP patients and 10 healthy controls.

### Data processing

The raw data were filtered and standardized using the tools available from Bioconductor version 3.7 [14], and the processed data were transformed into a gene expression matrix. The RMA (Robust multi-array Average) method [15] of affy packet was used to perform background correction, normalization and log_2_ transformation of the gene expression data. The differential expression analysis was performed using Limma (Linear Models for Microarray Data) package with empirical Bayesian method [16] to identify differentially expressed genes (DEGs) in ITP patients compared with healthy controls. The fold change more than 1.5 (FC > 1.5) and *P*-value less than 0.05 (*P* < 0.05) were considered as statistically significant differences.

### Enrichment analysis of DEGs

Gene ontology (GO) [17] and Kyoto Encyclopedia of Genes and Genomes (KEGG) pathway [18] enrichment analysis of DEGs were performed using Annotation, Visualization and Integrated Discovery (DAVID version 6.8, http://david.abcc.ncifcrf.gov/) [19]. The Gene Set EnrichmentAnalysis (GSEA) was used to perform GO terms enrichment analysis [20]. A *P* < 0.05 was considered statistically significant differences.

### Construction of protein-protein interaction network

Protein-protein interaction (PPI) network of the DEGs was constructed by searching against the online database STRING 11.0 (http://string-db.org) [21]. Subsequently, cytoHubba plug-in of Cytoscape software v3.9.0 [22] was used to screen the hub genes with the top 15 degree value in the network.

### Clinical samples collection

According to the diagnostic criteria of ITP recommended by American Society of Hematology 2019 guidelines [1,23], 30 chronic ITP patients admitted to Affiliated Cancer Hospital of Zhengzhou University from January 2020 to June 2020 were recruited in this study. Meanwhile, 26 sex- and age-matched healthy subjects were recruited as the control group. Characteristics of the ITP patients and controls enrolled in this study were shown in Table 1.

**Table 1.** Clinical characteristics of ITP patients and healthy controls

Items	Chronic ITP	Control
Number of subjects	30	26
Gender (male:female)	16:14	13:13
Median age (range) (years)	36.60 (19–51)	32.62 (18–55)
Median platelet count (range) (×10^9^/L)	8.500 (1–22)	-

The written informed consent was obtained from all individual participants. This study was approved by the Life Science Ethics Committee of Zhengzhou University (permit No. 2020–404-002 on 16 April 2020).

### Preparation of peripheral blood-derived T cells

According to a previous study [12], the T cells of heparin anti-coagulated blood from each subject were isolated. In brief, after collection of blood, the peripheral-blood mononuclear cells (PBMCs) were immediately separated by density gradient centrifugation. To prepare the total T cells, the magnetic activated cell sorter T-cell isolation kit (Miltenyi Biotech, Bergisch-Gladbach, Germany) was used for indirect isolation of untouched CD3^+^ T cells from the collected PBMCs, and then stored at −80°C for subsequent RNA and protein extraction.

### RNA isolation and quantitative reverse transcription PCR

Total RNA from T cells was extracted using Trizol reagent (Invitrogen, California, USA). The reverse transcription was performed using the Revert Aid First-Strand cDNA Synthesis Kit (Thermo scientific, Massachusetts, USA) according to the manufacturer’s instructions. Quantitative reverse transcription PCR (qRT-PCR) master-mix was prepared using SYBR® Premix Ex TaqTMII (Tli RnaseH Plus) (Takaro, Kyoto, Japan) and qRT-PCR analysis was performed using CFX96TM RealTime PCR Detection System (Bio-Rad, California, USA). The primer sequences used for qRT-PCR analysis were shown in Table 2. Each sample was repeated three times. The relative expression levels of genes were calculated based on 2^−ΔΔCt^ method [24]. GAPDH was used as an internal control.

**Table 2.** The primer sequences used for qRT-PCR analysis

Name	Forward (5’-3’)	Reverse (5’-3’)
GAPDH	TCAAGATCATCAGCAATGCC	CGATACCAAAGTTGTCATGGA
LCN2	GAGAACTTCATCCGCTTCTC	GATACACTGGTCGATTGGG
S100A8	CGTCTACAGGGATGACCTG	TTTCCTGATATACTGAGGACACTC
IL1R2	CGTCTGCACTACTAGAAATGC	GCAGGAAAGCATCTGTATTCTC
TLR4	GCCTTTTCTGGACTATCAAG	AATTTGAAAGATTGGATAAG
IFNG	TCCAAGTGATGGCTGAACTG	CTCTTCGACCTCGAAACAGC
CAMP	CACAGCAGTCACCAGAGGATTG	GGCCTGGTTGAGGGTCACT
CTSG	AAACACCCAGCAACACATCA	TATCCAGGGCAGGAAACTTG
ELANE	TCCACGGAATTGCCTCCTTC	TTGTGCCAGATGCTGGAGAG
LTF	AGAGCCTTCGTTTGCCAAGT	CATTTTGTGGCCTCGGGTTG
MMP9	TGTACCGCTATGGTTACACTCG	GGCAGGGACAGTTGCTTCT
MPO	GCAATGGTTCAAGCGATTCTT-	CGGTATAGGCACACAATGGTGAG
PADI4	CCATCCTGCTGGTGAACTGT	GAAGTCCTTGGGGGTCTTCG
S100A12	GGAGGGAATTGTCAATATC	ATCTTGATTAGCATCCAGG
S100A9	TTTGCTCCCCTTAATCCAGCC	CCTGGCAATTAGGGCAGTCG
SLPI	AATGCCTGGATCCTGTTGAC	AAAGGACCTGGACCACACAG

### Protein isolation and western blot analysis

The protein was extracted using the M-PER Mammalian Protein Extraction Reagent supplemented with Phosphatase Inhibitor Cocktail and 1X Halt Protease (Thermo Scientific, Massachusetts, USA). The protein concentration was evaluated using Pierce BCA Protein Assay Kit (Thermo Scientific, Massachusetts, USA).

The Blot system (Invitrogen, California, USA) was used to perform SDS-PAGE (10%) with MES running buffer, 4–12% Bis-Tris Plus gels, 1X LDS sample buffer and reducing agent. The protein was transferred to PVDF membranes (EMD Millipore, Massachusetts, USA). The membranes were blocked using Superblock T20 (PBS) Blocking Buffer (Thermo Fisher, Massachusetts, USA) and incubated using TBST (Beyotime Institute of Biotechnology, Jiangsu, China) containing 5% (w/v) skimmed milk powder at room temperature for 2 h, and then overnight with the primary antibody at 4°C.

Primary antibodies were diluted as follows: TLR4 1:1,000 (Cell Signaling Technology, Boston, USA), S100A8 1:1,000 (Cell Signaling Technology, Boston, USA) and β-actin 1:1,000 (Cell Signaling Technology, Boston, USA). The membranes were imaged using an Odyssey infrared imaging system (Li-COR Biosciences, Nebraska, USA). Immunoreactivity was determined using the enhanced chemiluminescence method (Pierce Chemical, Texas, USA). The experiment was repeated for three times.

### Statistical analysis

The data were expressed using mean ± standard deviation (SD). The difference between the two groups was tested by Student’s t-test. All analyses were performed using SPSS software 25.0 (IBM, New York, USA) [25]. A *P* < 0.05 was considered statistically significant difference.

## Results

### Identification of DEGs in peripheral blood-derived T cells in ITP patients

**Figure 1.**
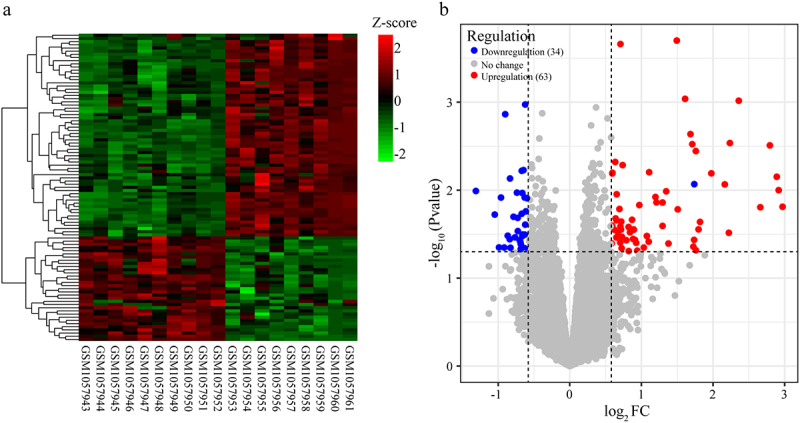
Identification of differentially expressed genes (DEGs) in ITP samples compared with healthy controls. (a) Heatmap showed expressed patterns of DEGs between the ITP samples and healthy controls based on Z-score. Red to green represented expression levels from low to high. (b) Volcano plot of DEGs in ITP patients compared with healthy controls. The Y axis represented log_2_ FC and the X axis represented -log_10_ (*P value*). Red square represented up-regulated genes and green circle represented down-regulated genes.

To identify the DEGs associated with ITP disease in peripheral blood-derived T cells, the DNA microarray data collected from a previous study were re-analyzed. A total of 20,186 genes were obtained and normalized (Table S1). Differential expression analysis showed that 97 DEGs, including 63 up- and 34 down-regulated were identified in ITP patients compared with healthy controls (Figure 1aandb, Table S2).

FC, fold change of ITP/control.

### Functional enrichment analysis of DEGs

**Figure 2.**
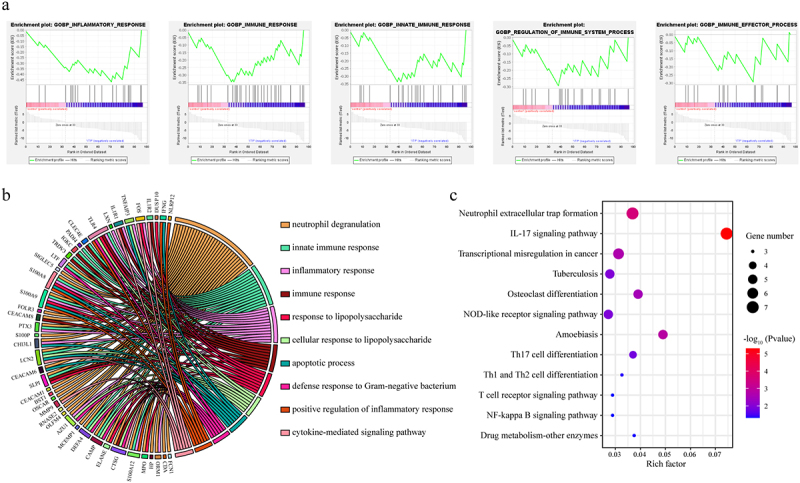
Functional enrichment analysis of differentially expressed genes (DEGs). (a) Gene ontology (GO) term enrichment analysis of DEGs using GSEA software. (b) GO term enrichment analysis of DEGs using DAVID bioinformatics resources. (c) Kyoto Encyclopedia of Genes and Genomes (KEGG) pathway enrichment analysis of DEGs. The size of circle represented the gene number. From blue to red represented the *P value* from low to high.

To explore the potential function of DEGs, the GO function and KEGG pathway enrichment analysis were performed. The results of GO analysis showed that the DEGs were associated with multiple immune-related terms, such as inflammatory response, immune response, and innate immune response (Figure 2aandb, Table S3). KEGG pathway analysis showed that there were 12 significantly enriched pathways (*P* < 0.05), including ‘Neutrophil extracellular trap formation’, ‘IL-17 signaling pathway’, and ‘Transcriptional misregulation in cancer’ (Figure 2c, Table S3).

### Construction of PPI network and identification of hub genes

**Figure 3.**
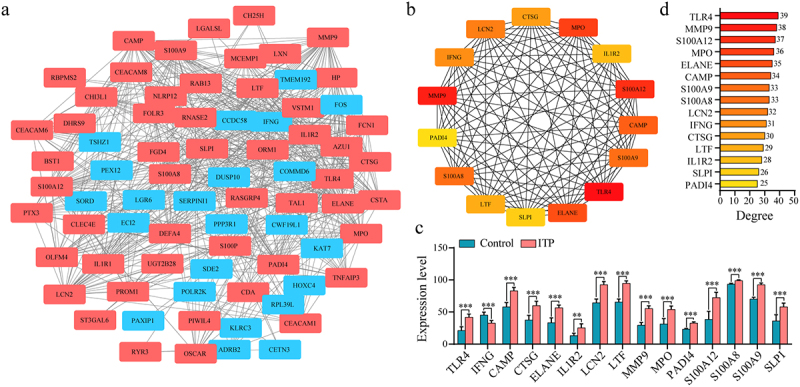
Construction of protein-protein interaction (PPI) network of differentially expressed genes (DEGs). (a) The PPI network generated in this study. Red rectangle represented up-regulated DEGs and green rectangle represented down-regulated DEGs. (b) Hub genes screened from protein-protein interaction network based on degree score. Orange to red represented the degree from low to high. (c) The expression levels of the 15 hub genes in DNA microarray data. (d) Histogram showed the degree of the 15 hub genes. **P < 0.01, *** *P* < 0.001 represented statistically significant difference.

To identify the hub genes involved in ITP, the PPI network of all DEGs was constructed. The network contained 76 nodes and 533 interactions (Figure 3a, Table S4). Subsequently, the top 15 genes ranked by degree value were selected as hub genes, including 14 significantly up-regulated DEGs (*TLR4, MMP9, S100A12, MPO, ELANE, CAMP, S100A8, S100A9, LCN2, CTSG, LTF, IL1R2, SLPI*, and *PADI4*) and one significantly down-regulated DEG (*IFNG*) (*P* < 0.05) (Figure 3bandc). Among these hub genes, *TLR4* had the highest degree score, indicating that the gene had the highest correlation with other genes in the network. In addition, the *S100* gene family members, including *S100A8, S100A9*, and *S100A12* displayed a high degree (Figure 3d).

### Validation of the hub genes by qRT-PCR

**Figure 4.**
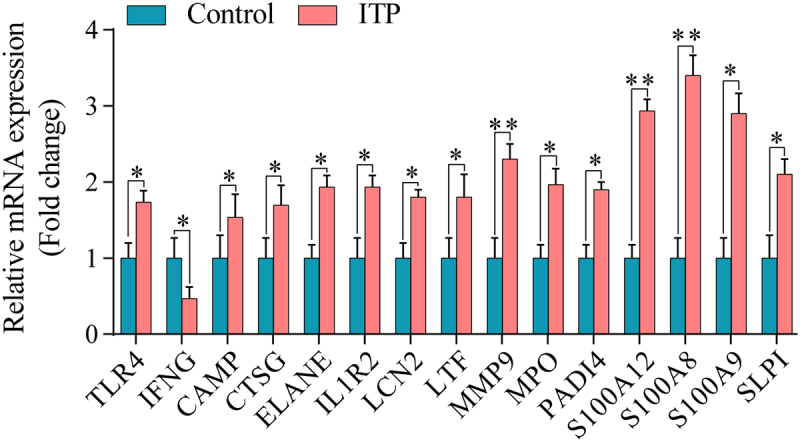
The mRNA levels of the 15 hub genes in ITP patients and healthy controls. **P < 0.01, ***P < 0.001 represented statistically significant difference.

To verify the reliability of DNA microarray dataset, the mRNA expression levels of 15 hub genes were measured by qRT-PCR. The results showed that 14 hub genes, including *TLR4, MMP9, S100A12, MPO, ELANE, CAMP, S100A8, S100A9, LCN2, CTSG, LTF, IL1R2, SLPI*, and *PADI4* were significantly up-regulated, while one hub gene *IFNG*, was significantly down-regulated in ITP patients compared with controls (*P* < 0.05) (Figure 4), which is consistent with the microarray analysis data. In addition, we found the *S100* family genes, including *S100A8, S100A9*, and *S100A12* had higher fold change than other hub genes (Figure 4), indicating that the *S100* genes play important roles in ITP disease.

### The protein levels of TLR4 and S100A8

**Figure 5.**
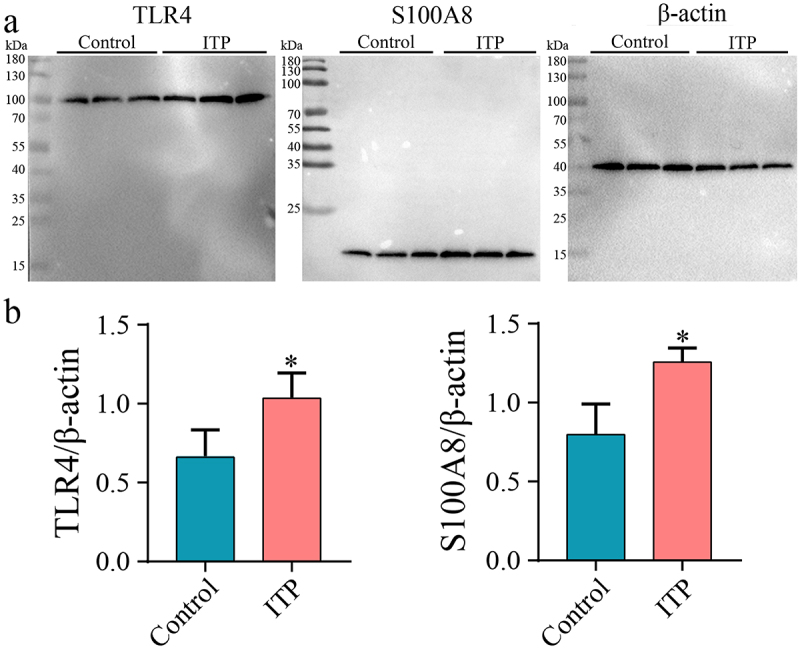
Determining the protein levels of *TLR4* and *S100A8*. (a) The protein levels of *TLR4* and *S100A8* in ITP patients and healthy controls. (b) Quantification of protein levels of *TLR4* and *S100A8*. *P < 0.05 represented statistically significant difference.

As *TLR4* exhibited the highest degree score in PPI network, and *S100A8* exhibited the largest fold change in qRT-PCR, the protein expression levels of these two genes were further verified by western blot analysis. The results showed that the protein levels of *TLR4* and *S100A8* were significantly up-regulated in ITP patients compared with controls (*P* < 0.05) (Figure 5 a and b).

## Discussion

In the present study, through bioinformatics analysis of microarray data, a total of 97 DEGs, including 63 up-regulated and 34 down-regulated were identified in peripheral blood-derived T cells in chronic ITP patients compared with healthy controls. PPI network analysis of all DEGs identified 15 top hub genes ranked by degree score, including 14 up-regulated and one down-regulated genes. Among these hub genes, *TLR4* had the highest score and *S100* family genes, including *S100A8, S100A9*, and *S100A12* had higher fold change than other hub genes, especially *S100A8* had the highest fold change in qRT-PCR. The protein levels of *TLR4* and *S100A8* were also significantly up-regulated in ITP patients.

Toll-like receptors (TLRs), as phylogenetically conserved receptors, can promote the transition of naive T cells to Th0, Th1, or Th2 phenotype, thereby regulating innate and adaptive immune responses [26,27]. Accumulating evidence has shown that *TLR4*, which expressed in various T cell subsets, including CD4^+^ T cells, CD8^+^ T cells, Tregs and natural killer (NK) T cells [28], is an important contributor to the development of multiple autoimmune diseases, such as systemic lupus erythematosus (SLE), rheumatoid arthritis (RA) [29], and experimental autoimmune encephalomyelitis (EAE). For instance, increased *TLR4* causes lupus-like disease and autoimmune glomerulonephritis [30,31]. RA can induce the expression of *TLR4* and thymoquinone ameliorates the disease by downer-gulating *TLR4* [32]. The expressed *TLR4* in T cells plays an essential role in EAE development [33]. Recently, a report demonstrated the knockout *TLR4* can inhibit thrombocytopenia and hemorrhage caused by Dengue virus in mice [34]. Reportedly, TLR4 may play a role through the TLR4-cytokine-CD4^+^ T lymphocyte cell pathway in the pathogenesis of ITP [35]. In this study, we found *TLR4* had the highest degree score in PPI network and was significantly up-regulated in T cells isolated from ITP patients, which further confirms the role of TLR4 in ITP.

The S100 family proteins, as the calcium-binding proteins, are the alarmins of multiple autoimmune and auto-inflammatory diseases, such as RA and SLE [36]. Some S100 family proteins, such as S100A8, S100A9, and S100A12, were found to regulate inflammation and proliferation in RA disease [37]. In RA and osteoarthritis patients, three S100 proteins, including S100A8, S100A9, and S100A12 are the most up-regulated biomarkers [38]. S100A8 and S100A9 are significantly induced by RA disease in inflammatory granulocytes and macrophages [39]. S100A12 facilitates the damage and erosion of joints and is associated with the pathological process of joint inflammation [40]. SLE can increase the levels of S100A8, S100A9, and S100A12 in serum and the combination of the three proteins can be used as biomarkers for lupus nephritis caused by SLE [41]. Sui et al proved that the plasma levels of S100A8 and SA100A9 were significantly increased in acute immune thrombotic thrombocytopenic purpura patients compared with healthy controls [42]. In the present study, we found that *S100A8, S100A9*, and *S100A12* had higher fold change compared with other hub genes, especially *S100A8* with the largest fold change in qRT-PCR. Furthermore, the protein level of *S100A8* was also significantly up-regulated in IPT patients than that in controls, indicating that *S100A8* played crucial roles in the pathological process of ITP disease.

In conclusion, through an integration approach of bioinformatics analysis and the clinical verification, two core genes, *TLR4* and *S100A8* were identified in peripheral blood-derived T cells in chronic ITP patients, which might be involved in the pathogenesis and progression of ITP. Further studies are needed to analyze the expression characteristics of *TLR4* and *S100A8* in different T cell subsets during the development of ITP. Collectively, the key genes identified here provided insights into the better understanding of ITP.

## Supplementary Material

Supplemental Material
